# Spirituality, Religious Diversity and Holistic Nursing Care in Nursing Education: An Exploratory Study Among Nursing Students in Italy

**DOI:** 10.3390/nursrep16040144

**Published:** 2026-04-17

**Authors:** Elisa Porcelli, Carla Murgia, Serena Caponetti, Gennaro Rocco, Alessandro Stievano, Ippolito Notarnicola

**Affiliations:** 1 San Filippo Negro Hospital/ASL Roma1, Catholic University of Sacred Heart, 00135 Rome, Italy; elisaporcellirm@libero.it (E.P.); carla.murgia@aslroma1.it (C.M.); 2 “A. Murri” Hospital, 63900 Fermo, Italy; serena.caponetti@gmail.com; 3 Centre of Excellence for Nursing Scholarship, OPI, 00131 Rome, Italy; genna.rocco@gmail.com; 4 Department of Clinical and Experimental Medicine, University of Messina, 98125 Messina, Italy; alessandro.stievano@gmail.com; 5 Department Medicine and Surgical, University of Enna “Kore”, 94100 Enna, Italy

**Keywords:** spirituality, spiritual care competence, nursing students, religious diversity, nursing education, holistic nursing

## Abstract

**Background:** Spirituality and religious diversity are increasingly recognized as essential components of holistic nursing care in global healthcare systems. However, their integration into undergraduate nursing education remains heterogeneous and often insufficiently structured, creating a gap between professional values and students’ preparedness to address spiritual needs in culturally diverse clinical environments. This study aimed to explore nursing students’ perceptions, attitudes, and perceived competencies regarding spirituality, religion, and spiritual care. **Methods:** A descriptive exploratory survey was conducted on a sample of 69 third-year nursing students (69.6% female; majority aged ≤24 years) enrolled in two universities in Rome, Italy. Data were collected between May and July 2025 using the Nursing Care and Religious Diversity Scale (NCRDS), consisting of 31 items. Statistical analyses included descriptive statistics, internal reliability analyses, group comparisons, and multivariate regression models. **Results:** Students showed moderate levels of attention to patients’ spiritual needs (mean = 3.11, SD = 0.88) and integration of spirituality into care practice, while high importance was attributed to spiritual care skills. University education was perceived as only partially adequate. Multivariate analyses showed that students’ personal spirituality is positively associated with the assessment of spiritual needs and the perception of competence, while exposure to contexts characterized by greater religious diversity is associated with a lower perception of preparedness. **Conclusions:** The results highlight a discrepancy between professional values and perceived operational skills, suggesting the need to systematically integrate spirituality and religious diversity into nursing curricula. These findings highlight the need for a structured integration of spirituality and religious diversity into nursing curricula through targeted educational strategies in order to strengthen students’ competencies and promote truly holistic and person-centered care.

## 1. Introduction

Spirituality represents an increasingly recognized component of assistance to the person, understood in its global, relational and existential dimension. In contemporary healthcare, spirituality is described as a resource through which individuals, patients and professionals attribute meaning to the experience of life, illness and care, regardless of adherence to specific religious traditions [1,2]. In this perspective, spirituality is not configured as an accessory element but as a transversal dimension capable of supporting the processes of adaptation, resilience and coping in contexts characterized by high care complexity.

Recent literature emphasizes how spirituality fosters connection with oneself, with others, and with what is perceived as meaningful or transcendent, helping to preserve the emotional and moral well-being of health professionals exposed to high care burdens [3,4]. In particular, in clinical environments with high emotional intensity, the spiritual dimension plays an important role in supporting the quality of the care relationship and in promoting a truly person-centered approach.

In the care pathway, spirituality manifests itself as a crucial element throughout life, acquiring specific relevance in times of crisis, vulnerability and transition. Recent studies show how, in situations of chronic illness, end of life or traumatic events, attention to the spiritual dimension contributes to improving the experience of care and the perception of dignity by patients [5,6]. At the same time, nurses recognize spirituality as an essential component of holistic care, although they often state difficulties in translating this awareness into concrete interventions.

In recent years, scientific interest in spirituality and spiritual care in nursing has grown significantly, with a steady increase in publications dedicated to training, skills, and related care practices. However, despite this development, numerous studies report a persistent discrepancy between the theoretical recognition of spirituality and its effective integration into educational and clinical settings [7,8]. This disconnect is particularly evident in university education courses, where spirituality is often treated in a marginal or unsystematic way.

Nurses, and in particular nursing students, work today in contexts characterized by growing cultural, religious and value diversity. This plurality requires advanced skills not only on a technical-clinical level but also on a relational, communicative and ethical level. Recent evidence suggests that the absence of adequate spiritual and religious literacy can generate uncertainty, avoidance or rigidity in care interactions, especially towards patients belonging to religious or cultural minorities [4,9].

In the educational field, several authors have highlighted how nursing curricula tend to privilege biomedical and organizational dimensions, relegating spirituality to sporadic or optional initiatives. Such an approach risks limiting the development of transferable skills in clinical practice and entrusting the management of the spiritual dimension exclusively to the personal sensitivity of the student or professional [2,10]. As a result, the quality of spiritual care may be uneven and dependent on individual factors rather than shared professional standards.

Recent studies in the field of nursing education underline the importance of integrating spirituality within structured, longitudinal and competence-oriented training models. Approaches based on experiential learning, simulation, and guided reflection have been associated with improved student preparedness and greater confidence in dealing with complex clinical situations involving existential and spiritual dimensions [3,5]. This evidence suggests the need to overcome predominantly theoretical teaching models, promoting a more authentic integration between training and practice.

In the light of these considerations, it is evident that spirituality and religious diversity represent strategic areas for the evolution of contemporary nursing education. However, significant gaps remain in the understanding of how nursing students perceive these dimensions and what factors influence the development of spiritual competencies during their university education. In particular, there is still limited empirical evidence on students’ preparedness to address spirituality and religious diversity in clinical settings, especially within increasingly multicultural healthcare environments. In particular, the empirical evidence relating to contemporary nursing education, characterized by a secular educational system and a growing cultural and religious heterogeneity, is still limited.

Through a quantitative exploratory design, the study contributes to bridging this knowledge gap and provides elements to guide the development of training programs more consistent with the complexity of contemporary care contexts.

Increasing cultural and religious diversity across healthcare systems has transformed the expected competencies of future nurses. Educational institutions are therefore challenged to prepare students capable of delivering inclusive, person-centered care that respects diverse belief systems. Understanding how nursing students perceive spirituality and their preparedness to address spiritual needs is essential to inform curriculum development aligned with global educational priorities. Unlike previous studies primarily focused on spiritual care competence alone, this study examines the interplay between personal spirituality, exposure to religious diversity, and perceived preparedness.

Therefore, this study aims to explore nursing students’ perceptions, attitudes, and perceived competencies regarding spirituality, religion, and spiritual care and to examine the factors associated with these dimensions within the Italian educational context.

## 2. Materials and Methods

### 2.1. Study Design

The present study was conducted using a cross-sectional survey design with an exploratory descriptive approach, aimed at analyzing the perceptions, attitudes and perceived skills of nursing students in relation to spirituality, religion and spiritual care in educational and clinical contexts. The choice of an exploratory approach was motivated by the complex and multidimensional nature of the phenomenon investigated, as well as by the relative scarcity of empirical evidence available the contemporary nursing education. This design allowed for obtaining a first systematic understanding of the students’ experiences and representations, without the intention of testing predefined causal hypotheses. The study was conducted and reported in accordance with the STROBE (Strengthening the Reporting of Observational Studies in Epidemiology) guidelines for observational studies in order to ensure transparency, completeness and accuracy in the description of the design, methods and results [11].

### 2.2. Study Context and Participants

The study involved students enrolled in the third year of the degree course in nursing belonging to two Roman universities, selected as representative of educational contexts characterized by high clinical exposure and growing cultural and religious diversity in the internship places. In particular, students from the Catholic University of the Sacred Heart, belonging to the training centers connected to the ASL Roma 1 and the San Filippo Neri Hospital, as well as to the Daughters of St. Camillus Institute-Father Luigi Tezza School, participated. Additional participants came from the “La Sapienza” University of Rome, with students enrolled in the G. Eastman Polyclinic and the Santo Spirito Hospital.

Third-year students were selected because they have already completed a substantial portion of both theoretical training and clinical placements, making them more likely to have been exposed to spiritual care situations. All students enrolled in the third year of the participating institutions were invited to participate. Participation was voluntary and anonymous, and no incentives were provided.

The decision to include multiple universities and clinics has made it possible to explore the perceptions of students in different organizational contexts, united, however, by a high complexity of care and a significant heterogeneity of the assisted population. All students enrolled in the third year in the universities involved were invited to participate in the study, without specific exclusion criteria, in order to obtain the widest possible representation of the educational experiences.

All third-year nursing students enrolled in the participating institutions were invited to take part in the study. Due to the involvement of multiple training sites and institutional settings, it was not possible to determine precisely the total number of students who received the invitation; consequently, a response rate could not be calculated. No formal exclusion criteria were applied in order to ensure a broad and representative inclusion of students’ educational experiences.

### 2.3. Data Collection

The data collection was carried out in a period between 19 May and 3 July 2025. Before the start of the study, the students were informed about the objectives of the research, the voluntary nature of participation and how the data will be processed. The questionnaire was distributed either during scheduled educational sessions or through institutional communication channels, depending on the organizational procedures of each participating site. Each participant was required to provide informed consent in writing. The complete anonymization of the data collected was guaranteed, and no information was acquired that could allow the direct or indirect identification of the participants.

The questionnaire was administered either in paper or electronic format, depending on the organizational procedures of each participating institution. The content, structure, and administration conditions of the questionnaire were identical across formats, ensuring consistency and minimizing potential methodological bias related to data collection mode.

Given the self-report nature of the questionnaire, a potential social desirability bias was considered during data interpretation. The average compilation time was low, favoring high adherence and reducing the risk of incomplete answers. At the end of the collection phase, the final sample consisted of 69 students who had fully completed the questionnaire.

Ethical considerations were addressed in accordance with the Declaration of Helsinki, and ethical approval was obtained from the Comitato Etico CECRI-Centro di Ricerca Clinica e Infermieristica (protocol code Prot. n. 8758/2024, approval date: 20 July 2024).

### 2.4. Detection Tool

The Nursing Care and Religious Diversity Scale (NCRDS) was used for data collection, a structured and anonymous tool consisting of 31 items, developed to explore nursing students’ perceptions in relation to spirituality, religion and religious diversity in care settings. The items are formulated as statements evaluated through five-point Likert-type scales, with increasing scores indicative of greater frequency, agreement or relevance attributed to the aspects investigated.

The NCRDS addresses a critical gap in the measurement of nursing students’ preparedness to provide spiritually and culturally responsive care, particularly within healthcare systems characterized by increasing religious pluralism.

The questionnaire allows a multidimensional evaluation of the phenomenon through different conceptual areas. A first part collects basic socio-demographic information, useful for the description of the sample and for comparative analyses. A second area explores the educational and clinical context of the students, including information relating to the university location and internship locations. The following sections are focused on spiritual assistance to others, investigating the frequency with which students assess the spiritual needs of patients, their willingness to integrate these aspects into care practice, and perceived difficulties. Finally, a part of the questionnaire explores spiritual and religious expression, including items related to opinions on spirituality, the importance attributed to skills in the field of spiritual assistance and the perception of the adequacy of university education.

Each item was rated on a five-point Likert scale ranging from 1 (‘strongly disagree’ or ‘never’) to 5 (‘strongly agree’ or ‘always’), depending on the nature of the item. Previous studies using similar instruments have reported acceptable psychometric properties, and in the present study, internal consistency ranged from 0.74 to 0.88, indicating good reliability.

The NCRDS was developed to explore nursing students’ perceptions of spirituality, religion, and religious diversity in care contexts, addressing a gap in available measurement tools within nursing education. Although the instrument has not undergone full psychometric validation in previous studies, its internal consistency was assessed in the present sample, showing satisfactory reliability coefficients. This study therefore represents an initial empirical application of the instrument, and further research is recommended to confirm its factorial structure and psychometric properties in larger and more diverse populations.

### 2.5. Data Management and Analysis

Prior to the statistical analysis, a preliminary check of the completeness and consistency of the data was carried out in order to identify any input errors or missing answers. The datasets were then exported and analyzed using the statistical software SPSS, version 22.

The analysis of the data was divided into several phases. First, descriptive statistics were used to synthesize the socio-demographic characteristics of the sample and to describe the distribution of responses to individual items and aggregate scales. Categorical variables were expressed as absolute frequencies and percentages, while continuous variables were described by mean and standard deviation.

Subsequently, composite scales were constructed from groups of conceptually similar items in order to explore dimensions such as the frequency of assessment of spiritual needs, attitudes towards spirituality in care, the importance attributed to spiritual assistance skills and the perceived adequacy of university education. For each scale, internal reliability was assessed by calculating the Cronbach coefficient.

Inferential analyses were also conducted to explore any differences between groups in relation to the main socio-demographic variables. Finally, in order to identify the factors associated with the main dimensions of interest, multivariate regression models were constructed, including socio-demographic and contextual variables considered relevant in relation to the objectives of the study.

Given the exploratory nature of the study, the sample size was considered adequate to identify preliminary trends and associations. Similar exploratory studies in nursing education have used comparable sample sizes to generate initial evidence and inform future larger-scale research.

Group comparisons were performed using independent samples *t*-tests. Statistical significance was set at *p* < 0.05. Prior to inferential analyses, the assumptions of normality and homogeneity of variance were assessed to ensure the appropriateness of the applied statistical tests.

## 3. Results

The final sample of the study consisted of 69 third-year students of the Degree Course in Nursing from two Roman universities. The majority of the participants were female, mainly under the age of 25 and of Italian nationality. The complete socio-demographic characteristics of the sample, including gender, age group, marital status, previous education and internship location, are shown in Table 1.

As regards the environmental and institutional dimension, a significant part of the students reported the presence of Christian religious symbols in university and internship contexts, while the presence of signs or references to other religions was generally limited. Similarly, the availability of prayer spaces accessible to people of different religious affiliations has been reported in an uneven way between the different internship sites, suggesting an unsystematic management of the religious dimension in training and care contexts.

The descriptive analysis of the dimensions explored by the Nursing Care and Religious Diversity Scale highlighted an overall moderate level of attention to spirituality and religion in care practice. In particular, the frequency with which students reported exploring patients’ spiritual and religious needs, including asking for information related to beliefs, religious practices, and spiritual needs, showed an average score of 3.11 (SD = 0.88) on a five-point scale. This indicates a moderate level of engagement in the assessment of patients’ spiritual needs.

Attitudes towards spirituality in care recorded an average value of 3.29 (SD = 0.74), indicating a generally positive but not fully consolidated attitude. Similarly, opinions on spirituality as a component of the care relationship and the well-being of the person showed an average score of 3.25 (SD = 0.80), confirming a theoretical awareness of the value of the spiritual dimension, not always accompanied by full integration into care practice.

A higher average score emerged in relation to the importance attributed to nursing skills in spiritual care, with a value of 3.47 (SD = 0.83). This reflects higher mean scores in the importance attributed to spiritual care skills. The perceived adequacy of university education with respect to the development of skills in the spiritual field showed an average value of 3.25 (SD = 0.83), suggesting a preparation considered sufficient but not fully satisfactory. The complete descriptive statistics of the dimensions explored and the related internal confidence indices are shown in Table 2.

The internal reliability analyses of the scales showed Cronbach’s alpha values between 0.74 and 0.88, indicating a good internal consistency of the considered dimensions and supporting the use of the aggregate scales in subsequent inferential analyses.

As for comparisons between groups, the analysis by gender showed a statistically significant difference in opinions on spirituality, with higher average scores among female students than male students (*p* = 0.010).

However, these findings should be interpreted with caution due to the unequal distribution between groups and the relatively small size of the male subgroup (*n* = 19), which may limit the statistical robustness of these comparisons. Therefore, these results should be considered exploratory and hypothesis-generating rather than confirmatory.

Although not all differences reached statistical significance, consistent trends emerged indicating higher average values in the female group also with regard to the importance attributed to spiritual care skills and the perception of educational adequacy. The detailed results of the comparisons by gender are shown in Table 3.

Multivariate analyses allow us to explore the factors associated with the frequency of assessment of spiritual needs and the perception of adequacy of university education. In the regression model for the frequency of spiritual exploration, a higher frequency of religious practice and greater importance attributed to religion in daily decisions were significantly associated with a higher involvement in the spiritual dimension of caregiving. Conversely, greater exposure to patients from non-European countries was associated with a lower frequency of spiritual needs assessment.

A similar pattern emerged in the model that analyzed the perceived adequacy of training. Also in this case, the frequency of religious practice and the importance attributed to religion were positive predictors, while exposure to patients with religious affiliations other than Christian was associated with a lower perception of preparedness. The regression models explained a moderate share of the variance of the outcomes considered, suggesting that spiritual skills and attitudes are influenced by personal, educational and contextual factors. The complete results of the regression analyses are shown in Table 4.

Overall, the results indicate that although nursing students recognize the value of spirituality and spiritual care as fundamental components of holistic care, uncertainties and critical issues remain related to educational preparation and the management of religious diversity in clinical settings. These difficulties emerge particularly clearly precisely in contexts characterized by greater cultural and religious complexity.

## 4. Discussion

The present study explored nursing students’ perceptions, attitudes, and perceived competencies regarding spirituality, religion, and spiritual care within a university educational environment shaped by growing cultural and religious diversity. Overall, the results outline a student population aware of the importance of the spiritual dimension in care but which encounters significant difficulties in translating this awareness into structured operational skills, particularly in clinical contexts with high care complexity.

The findings confirm a moderate level of attention to spirituality and spiritual care among nursing students, in line with previous literature. This figure appears to be in line with recent evidence that describes how nursing students recognize the relevance of spirituality on the value level without, however, feeling fully prepared to deal with it in daily clinical practice [12,13]. In particular, the exploration of patients’ spiritual needs emerges as unsystematic and often subordinate to clinical priorities, confirming a predominantly reactive rather than proactive approach.

A central element that emerged from the study concerns the discrepancy between the high importance given to spiritual assistance skills and the perception of insufficient university preparation. Students recognize spirituality as an integral part of holistic care and the professional nursing role, but report training perceived as fragmented and poorly integrated into clinical pathways. Recent studies conducted in different European contexts confirm that spirituality is often addressed implicitly or marginally in curricula, without clear educational objectives or tools for assessing the skills acquired [14,15].

The high internal reliability of the dimensions explored supports the robustness of the analyzed constructs and strengthens the interpretation of inferential results. In particular, the conceptual clarity of the dimensions related to views on spirituality and the importance of skills suggests that these areas are perceived as distinct and significant domains also in the student population. This finding is consistent with recent theoretical contributions that underline the need to formally recognize spirituality as a specific professional competence and not as a mere personal quality of the individual nurse [16,17].

The differences that emerged in comparisons by gender, with higher scores among female students in opinions on spirituality, are consistent with recent studies that report greater female sensitivity to the relational, emotional and existential dimensions of care. These differences seem to reflect socio-cultural and formative dynamics rather than biological factors, suggesting the influence of educational and professional models that encourage greater expression of the relational dimension in women [13].

Multivariate analyses showed that students’ personal spirituality is associated with greater attention to patients’ spiritual needs and a higher perception of preparedness. This result confirms what has been reported by recent studies, according to which, in the absence of structured formation, personal spirituality tends to represent the main resource that students draw on to deal with the spiritual dimension of care [16,17]. However, this dynamic entails the risk of a marked heterogeneity in care practices and an excessive dependence on individual factors.

Particularly critical is the data relating to the negative association between exposure to patients belonging to non-majority religious contexts and the perception of spiritual competence. This finding suggests that religious diversity, if not adequately supported by educational and reflective tools, can generate insecurity, avoidance, or reduced exploration of spiritual needs. Recent evidence indicates that the lack of structured intercultural and spiritual competences can turn diversity into a source of educational stress rather than an opportunity for learning and professional growth [12,15].

Taken together, the results confirm that spirituality is recognized by students as an essential dimension of nursing but remains poorly structured on an educational and operational level. This discrepancy risks translating, in professional practice, into a reduction in the holistic approach and a lower ability to respond to the spiritual needs of patients belonging to different cultural and religious contexts. In light of this, the need emerges to rethink training models in nursing, promoting a systematic integration of spirituality and religious diversity into curricula, in line with the needs of contemporary care contexts.

These findings reflect trends reported across different international contexts, reinforcing the global relevance of integrating spirituality into nursing education.

From an educational perspective, these findings highlight the need to implement structured and competency-based approaches to spiritual care within nursing curricula. Educational strategies such as reflective learning, simulation-based training, and case-based discussions may support the development of students’ ability to address spiritual and religious needs in clinical practice. Furthermore, integrating spirituality into clinical training pathways and assessment frameworks could contribute to reducing the gap between theoretical knowledge and practical application.

### 4.1. Limitations of the Study

Data were collected using a self-assessment tool, with the inherent risk of social desirability bias. The present study has some limitations that must be considered in the interpretation of the results. Firstly, the exploration and transversal design does not allow establishing causal relationships between the variables analyzed but only allows identifying associations and emerging patterns. Consequently, the results should be read as indicative of potential trends and relationships, which need further investigation through longitudinal studies or experimental designs.

A further limitation is represented by the size of the sample, consisting of 69 nursing students from two Roman universities. Although the sample is adequate for the exploratory objectives of the study, it limits the generalizability of the results to other university or geographical contexts. In addition, the prevalence of students of Italian nationality and the uneven distribution by gender may have influenced some of the associations observed, particularly those related to views on spirituality and spiritual assistance.

Furthermore, the inclusion of participants from only two higher education institutions may limit the generalizability of the findings to other academic or geographical contexts.

Data were collected using a self-assessment tool, with the inherent risk of social desirability bias. Students may have provided answers geared toward what they felt was professionally or ethically appropriate, rather than fully reflecting their own practices or beliefs. This limitation is particularly relevant when investigating sensitive constructs such as spirituality and religion, which are strongly influenced by personal values and cultural norms.

A further aspect to consider concerns the measurement of clinical experience and exposure to religious diversity, assessed through self-reported items. This modality may not comprehensively capture the complexity of the students’ experiences during the internship, nor the quality of interactions with patients belonging to different religious and cultural traditions.

Finally, although the Nursing Care and Religious Diversity Scale showed good internal reliability in the present sample, the study did not have as its primary objective the psychometric validation of the instrument. Therefore, future research should further investigate the factor structure and psychometric properties of the scale in larger and more diverse samples.

Despite these limitations, the study provides relevant contributions to the understanding of nursing students’ perceptions and skills in the field of spirituality and spiritual care, offering useful indications for the development of targeted educational interventions and for the design of future research in this area.

### 4.2. Research Implications

The results of the present study indicate the need to develop further lines of research aimed at understanding more deeply how spirituality and religious diversity can be effectively integrated into nursing education. In particular, the recent literature emphasizes the urgency of overcoming isolated descriptive approaches, promoting research programs capable of systematically linking education, clinical practice, and measurable educational outcomes [18,19]. In this perspective, longitudinal studies appear particularly suitable for analyzing the evolution of students’ spiritual skills during their university career and in the transition to professional practice.

A priority area of in-depth study concerns the evaluation of the effectiveness of structured educational interventions aimed at the development of spiritual skills. Recent contributions suggest that training models based on clinical simulation, reflective learning and the use of complex scenarios can foster greater integration between theoretical knowledge, attitudes and practical skills [20,21]. Experimental or quasi-experimental studies could help to identify which teaching strategies are most effective in supporting students’ preparedness in addressing the spiritual needs of patients in multicultural contexts.

Further research should deepen the conceptual distinction between personal spirituality, religiosity, and professional competence in spiritual counseling. The most recent literature highlights how a lack of clarification of these constructs can hinder the development of reliable and comparable assessment tools, limiting the possibility of building educational models based on observable competences [22,23]. In this sense, methodological studies aimed at validating specific tools for the nursing student population represent a further relevant line of research.

Particular attention should be paid to the study of students’ experiences in clinical contexts characterized by high religious and cultural diversity. The results of the present study suggest that such contexts may be perceived as a source of uncertainty and discomfort, rather than as learning opportunities. Qualitative and mixed-method approaches could explore in depth the relational dynamics, the emotions experienced and the adaptive strategies adopted by students, providing useful indications for designing more sensitive and contextualized training interventions [24,25].

A further line of research concerns the role of the institutional environment and the organizational context in supporting the development of spiritual skills. Recent studies suggest that the support of clinical tutors, organizational culture, and educational policies of academic institutions significantly influence students’ willingness to integrate spirituality into care practice [26,27]. Comparative analyses between different educational systems and national contexts could contribute to identifying organizational models favorable to the development of competent spiritual care.

Finally, future research should include larger and culturally diverse samples in order to improve the generalizability of the results and foster international comparison. The integration of quantitative and qualitative approaches, together with the use of standardized and culturally adapted tools, could contribute to the construction of a more solid evidence base, capable of guiding educational policies and training strategies in nursing [19,27].

Future studies should also specifically investigate the effectiveness of targeted educational strategies, such as simulation-based training, reflective learning, and case-based approaches, in enhancing spiritual care competencies among nursing students.

## 5. Conclusions

The present study explored the perceptions and perceived skills of nursing students in relation to spirituality and spiritual care, highlighting how this dimension is widely recognized as an integral part of nursing care but not yet fully consolidated at the educational level. Students attribute a high importance to spiritual care while reporting a university preparation perceived as only partially adequate to deal with the complexity of clinical contexts.

The results show a discrepancy between professional values and perceived operational skills, suggesting that spirituality is addressed in an unsystematic way and often subordinated to other care priorities, especially in contexts characterized by greater religious diversity. In such situations, the lack of adequate training tools can result in uncertainty and a reduction in professional action.

Overall, the study emphasizes the need to integrate spirituality and religious diversity into nursing curricula through structured and competency-based educational approaches, in order to strengthen students’ preparedness and promote truly holistic and person-centered care.

## Figures and Tables

**Table 1:** Socio-demographic characteristics of the sample.

Variable	*n*	%
**Gender**		
Male	19	27.5
Female	48	69.6
Non-binary	2	2.9
**Age**		
≤24 years old	49	71.0
25–29 years	12	17.4
30–34 years	5	7.2
≥35 years old	3	4.4
**Marital status**		
Single	57	82.6
Married	7	10.1
Cohabitant	3	4.3
Separated/Divorced	2	2.9
**Nationality**		
Italian	66	95.7
Other	3	4.3

**Table 2:** Descriptive statistics and reliability of the size of the NCRDS.

Size	Item Number	Mean	SD	Range	Cronbach’s α
Frequency of Spiritual Needs Assessment	7	3.11	0.88	1–5	0.78
Attitudes towards spirituality in care	6	3.29	0.74	1–5	0.74
Importance given to spiritual counseling skills	5	3.47	0.83	1–5	0.86
Views on spirituality	8	3.25	0.80	1–5	0.88
Perceived adequacy of university education	4	3.25	0.83	1–5	0.83

**Table 3:** Comparisons by gender on the main dimensions of spirituality and spiritual assistance.

Size	Males (*n* = 19) Mean ± SD	Females (*n* = 48) Mean ± SD	t	*p*
Frequency of Spiritual Needs Assessment	3.09 ± 0.85	3.16 ± 0.88	−0.27	0.788
Attitudes towards spirituality in care	3.05 ± 0.76	3.38 ± 0.72	−1.39	0.178
Importance of Spiritual Counseling Skills	3.17 ± 0.82	3.58 ± 0.82	−1.72	0.096
Views on spirituality	2.87 ± 0.77	3.41 ± 0.78	−2.71	**0.010**
Perceived appropriateness of training	2.93 ± 0.79	3.40 ± 0.83	−1.97	0.059

**Table 4:** Multivariate regression analysis: factors associated with spiritual assessment frequency and educational adequacy.

Independent Variables	Frequency of β Spiritual Needs Assessment (95% CI)	*p*	Perceived Adequacy of β Training (95% CI)	*p*
Gender (female vs. male)	0.07 (−0.32; 0.46)	0.718	0.18 (−0.19; 0.55)	0.337
Age	−0.05 (−0.18; 0.07)	0.406	−0.04 (−0.17; 0.09)	0.536
Frequency of religious practice	0.31 (0.12; 0.50)	0.002	0.29 (0.10; 0.48)	0.004
Importance of religion in everyday decisions	0.27 (0.09; 0.45)	0.004	0.24 (0.05; 0.43)	0.014
Exposure to non-European patients	−0.22 (−0.40; −0.04)	0.017	−0.25 (−0.44; −0.06)	0.009
Model R^2^	0.38		0.33	

## Data Availability

The data supporting the findings of this study are available from the corresponding author upon reasonable request. The data are not publicly available due to privacy and ethical considerations.

## Public Involvement Statement

No public involvement in any aspect of this research.

## Guidelines and Standards Statement

This manuscript was prepared in accordance with the STROBE (Strengthening the Reporting of Observational Studies in Epidemiology) guidelines for observational research [11].

## Abbreviations

The following abbreviations are used in this manuscript:

NCRDS	Nursing Care and Religious Diversity Scale
SPSS	Statistical Package for the Social Sciences
SD	Standard Deviation
CI	Confidence Interval
STROBE	Strengthening the Reporting of Observational Studies in Epidemiology
